# Stem cell systems informatics for advanced clinical biodiagnostics: tracing molecular signatures from bench to bedside

**DOI:** 10.3325//cmj.2013.54.319

**Published:** 2013-08

**Authors:** Randolph S. Faustino, D. Kent Arrell, Clifford D.L. Folmes, Andre Terzic, Carmen Perez-Terzic

**Affiliations:** 1 Division of Cardiovascular Diseases, Departments of Medicine, Molecular Pharmacology and Experimental Therapeutics, Mayo Clinic, Rochester, MN, USA; 2 Physical Medicine and Rehabilitation, Mayo Clinic College of Medicine, Rochester, MN, USA

## Abstract

Development of innovative high throughput technologies has enabled a variety of molecular landscapes to be interrogated with an unprecedented degree of detail. Emergence of next generation nucleotide sequencing methods, advanced proteomic techniques, and metabolic profiling approaches continue to produce a wealth of biological data that captures molecular frameworks underlying phenotype. The advent of these novel technologies has significant translational applications, as investigators can now explore molecular underpinnings of developmental states with a high degree of resolution. Application of these leading-edge techniques to patient samples has been successfully used to unmask nuanced molecular details of disease vs healthy tissue, which may provide novel targets for palliative intervention. To enhance such approaches, concomitant development of algorithms to reprogram differentiated cells in order to recapitulate pluripotent capacity offers a distinct advantage to advancing diagnostic methodology. Bioinformatic deconvolution of several “-omic” layers extracted from reprogrammed patient cells, could, in principle, provide a means by which the evolution of individual pathology can be developmentally monitored. Significant logistic challenges face current implementation of this novel paradigm of patient treatment and care, however, several of these limitations have been successfully addressed through continuous development of cutting edge *in silico* archiving and processing methods. Comprehensive elucidation of genomic, transcriptomic, proteomic, and metabolomic networks that define normal and pathological states, in combination with reprogrammed patient cells are thus poised to become high value resources in modern diagnosis and prognosis of patient disease.

Disease anticipation prior to symptomatic presentation provides multiple pre-emptive opportunities for clinical management. Palliative or curative effectiveness is enhanced by early detection of disease-promoting factors, as early stage treatment can preclude catastrophic pathological progression. Translationally relevant risk factors can be identified through techniques that interrogate and assess biological marker status (1), where methods such as gene overexpression, peptide fragment enrichment, and metabolite concentration can be respectively used to diagnose diseases such as clinical carcinoma (2), cardiovascular pathologies (3), and diabetes (4).

Distinctions among high throughput methodologies confer specific advantages and disadvantages that determine efficiency and application of genomic, proteomic, or metabolomic approaches. For example, genes validated from tissue biopsies and measurement of glucose uptake with the glucose analogue tracer 18F-fluoro-2-deoxyglucose are among the tools that oncologists rely upon to diagnose and manage tumors (5). Experimental confirmation of gene expression differences in normal vs tumor samples can then be used to determine tumor aggressiveness and provide prognosis and susceptibility profiles (6).

Differential gene expression provides direct indicators of genetic background and transcriptome reprogramming as a consequence of evolving disease, but cannot provide information regarding dynamic functional changes. Peptide screening, a hallmark of proteomic analyses, can be employed to obtain this type of information (7,8). For example, palliation of cardiopathology is more effective when combined with a proteomic strategy to identify peptide fragments released into patient serum as a result of cardiac dysfunction (9,10).

Advantages of genomic/transcriptomic and proteomic screens provide relevant data for clinical management of disease, though the techniques themselves require several days to weeks for quality-controlled processing and data analyses. Metabolic screening offers a distinct advantage in that samples can be assayed to provide feedback on disease states with greater celerity than the “-omic” approaches described previously. Though the prototype for metabolite analysis is glucose monitoring used in diabetes management (11), other examples of clinical metabolomics exist (12,13). Significantly, stabilization and processing of evanescent metabolites for precise and accurate measurements of metabolic states in diseased vs non-diseased patients is critical to all metabolomic profiling approaches.

These techniques allow deep interrogation of the molecular complement that comprises the functional background of a cell or tissue sample. Clinical application of these approaches gains significant leverage when combined with a pluripotent cell platform that can provide readouts of the molecular origin of disease phenotype.

## The post-genomic era and stem cells

Current generation high throughput technology, originally developed to address rapid high volume sequencing needs (14), has galvanized genomic, transcriptomic, proteomic, and metabolomic platform development. Depth and resolution of biomarker composition analysis has increased significantly, which has necessitated development of commensurate *in silico* methods. Indeed, bioinformatic approaches that employ systems biology principles to parse multidimensional biodata extracts multiple levels of integrated information, and application of such techniques to targeted stem cell populations may offer novel modalities of advanced pathology diagnosis.

Stem cells possess a unique potential to anticipate disease phenotypes as they harbor the fundamental molecular baseline that gives rise to genomic content and its derived transcriptomic, proteomic, and metabolomic strata. Interrogation of these “-omic” layers provides rich data to deconvolute systems biology of developmental programs, elucidation of which is critical to understanding clinical pathology etiology. Molecular cartography of pluripotent disease-prone backgrounds would facilitate pre-emptive diagnosis through comparison of healthy wild type templates with diseased states (3,15,16), as well as offer a tool for dynamic prognosis to track changes concomitant with pathological progression or assessment of response to therapeutic intervention (3). While a variety of stem cells offer specific clinical advantages, pluripotent stem cells may be ideal candidates for patient applications (17,18).

Embryonic stem (ES) cells can be harvested and profiled to establish a molecular baseline for comparison to diseased genomes. Recent data indicate that in addition to patient-donated material, patient-specific ES cells can be successfully cloned for potential therapeutic applications (19). Partly in an attempt to circumvent moral, legal, and ethical disputes associated with human ES studies, discovery and development of a molecular algorithm to reprogram cells from a committed fate to a pluripotent state (20) may provide a viable alternative to, and significant advantages over, ES cells in development of systems biology strategies for comprehensive disease resolution spanning embryonic to differentiated phenotypes (21). Generation and establishment of disease- and patient- specific embryonic stem cell lines is valuable for its potential to provide individualized experimental cell models, where tailored experimental design, with a focus on personalized therapeutic strategies, can be performed (22). Induced pluripotent stem (iPS) cells, as part of an advanced disease management strategy, have the potential to advance clinical diagnostics, though several obstacles face implementation of this strategy.

Full equivalency of induced compared to developmentally derived ES cells must be demonstrated to employ reprogrammed cells as proxies for a naturally occurring primordial baseline (23). Indeed, though genetic and epigenetic variation between stem and iPS cells confers a difference in tumorigenic potential (24), extraction of a conserved molecular signature may circumvent limitations associated with genetic and epigenetic heterogeneity (17,25). Furthermore, restriction of bioinformatic interrogation to iPS cell lines derived from the same patient can be used to limit influence of epigenetic variation among samples. In combination with quality control measures to ensure consistent cell culture technique and microenvironment exposure that may otherwise lead to respective chromosomal segregation and epigenetic changes (26,27), preparation of human iPS cell lines for clinical diagnostic applications may be feasible.

Systems-wide biodiagnostics, in which entire molecular complements can be assayed, provides an opportunity to comprehensively quantify molecular elements that underlie normal as well as clinically dysfunctional development. Current advances in cellular reprogramming methodology, along with the advent of high throughput technology and advanced bioinformatic computational approaches, presents a unique confluence of molecular tools and techniques by which diseased cells can be reprogrammed to a primordial state (28), then scrutinized for signature elements that segregate them from normal phenotypes. Thus, comprehensive bioinformatic dissection of phenotypic regressed ES cells offers opportunities to discover novel molecular targets for early intervention or palliation in advance of clinical manifestation, that may be found at genomic, transcriptomic, proteomic, and metabolomic levels, or any combination thereof.

## Genomics

The genomic, transcriptomic, proteomic, and metabolomic elements of systems biology approaches provide biological information unique to each layer of the molecular network, and a variety of specialized techniques exist to interrogate each. From a molecular network ontogeny perspective, the genome is the origin of subsequent transcriptomes, proteomes, and metabolomes, and is a useful starting point for a discussion regarding roles the genome has played in translational diagnosis.

The genome is the molecular blueprint composed of genic and non-genic sequences that ultimately determine organismal phenotype (29), and the advent of whole genome sequencing has catapulted the field forward (30), as well as given rise to new specialties (31). This advance has spurred a renaissance of clinical genetics, as present genetic counseling techniques are based on reductionist “one gene, one disease” strategies, a paradigm which has successfully guided identification of the genetic basis for many clinically relevant diseases. Resolution of polygenic pathologies, as well as disease progression impacted by heritable epigenetic modifications, however, cannot be effectively addressed by this diagnostic modality. To resolve the problem of multigene and/or epigenetically triggered pathology, genome-wide association studies (GWAS) have been used to identify and define multiple susceptibility loci correlated with disease presentation (32). This approach is used to bioinformatically mine complex genomic data sampled from large populations to identify key genes that associate with disease. Paradoxically, the large sample sizes that provide robustness to a GWAS approach prevents direct application toward personalized therapeutic approaches, a limitation that must be considered when evaluating genomic diagnostic technologies for individualized patient application.

Implementation of genome-wide analyses to stem cell (re)programming in the context of therapeutic application has yielded a rich body of novel data that provides details on changes in genomic regulatory elements, epigenomic landscape transitions, and three-dimensional chromatin shifts critical to the process of phenotype (re)acquisition (28,33). These levels of genomic complexity imposed can now be resolved with high precision using modern methodology (34) to facilitate fine resolution of the full genomic blueprint, critical for translational application.

## Transcriptomics

The transcriptome is the full complement of RNA produced in response to signaling cues processed by, and transcribed from, the underlying genome, and technology and methods employed for genomic deconstruction are applicable to transcriptome resolution. Comprehensive transcript analysis is an attractive option for biomarker identification, as panels of differentially expressed genes (DEGs) are used to establish indices of disease progression (35). Prioritized gene lists can be further analyzed for gene ontology enrichment and bionetwork analyses to respectively identify and quantitate the molecular gestalt underlying normal or diseased phenotype progression (36).

Transcriptome deconvolution has been used to identify contributions of specific genes during the process of somatic cell reprogramming (37), and a variety of criteria unique to high throughput RNA analysis that derive from diverse RNA heterogeneity (38) play critical roles in elucidating transcriptome dynamics of differentiation. An essential characteristic of the transcriptome is (auto)regulation facilitated by subtle intra-RNA dynamics (39,40), however interactions of regulatory non-coding RNA with molecular targets can be parsed using specific bioinformatic resources. Fate acquisition is driven by splice variance that occurs as a result of differential mRNA processing, and distinguishing these permutations from variations in background noise requires intensive computational resources (41). Development-dependent isoform switching presents another critical variable to temporal resolution of the transcriptome assembly during differentiation/reprogramming (42,43). In addition, high degrees of post-transcriptional regulation conferred by microRNA and long ncRNA mandates novel biostatistical models and *in silico* approaches to properly resolve transcriptome dynamics of fate commitment/reversion (44,45).

Ultimately, deeper data sets empower *in silico* tools, and is key to modern diagnostic techniques that employ next generation sequencing to leverage rich genomic and transcriptomic content against clinical diseases in order to facilitate high resolution etiology definition (46). Paired with quality biobank sample acquisition and leading-edge techniques for cell isolation and reprogramming, continuous development of high quality, publicly available computational tools will significantly advance transcriptome analysis, and will refine transcriptome interrogation as a modern tool for assaying gene expression dynamics associated with clinical pathologies.

## Proteomics

The proteome consists of all proteins expressed by a genome in a defined cell or tissue at a particular time, whereas proteomics comprises an array of techniques for studying expression, abundance, structure (including post-translational modifications) and function, including their physical and functional interactions (3). Modern proteomic approaches involve high throughput protein separation and processing followed by mass spectrometry for peptide and protein identification, either as intact entities or as peptide fragments, defined respectively as top-down and bottom-up proteomics (3). As proteins form the molecular machinery of the cell, alterations in their abundance and activity translate into detectable changes in other “-omic” strata, such as epigenetic modifications, mRNA abundance or differential splicing, and altered metabolite levels. Thus, comprehension of stem cell proteomes and their dynamics may provide detailed systems understanding of pluripotency, how it differs from somatic cell states, and may yield important clues into mechanistic understanding of progenitor cells for therapeutic and diagnostic applications.

Proteomic studies have increased our understanding of protein complexes and dynamics contributing to cell fate determination, of cell state transitions during development and reprogramming cells to pluripotency, and of the extensive molecular impact mediated by disease-targeted stem cell-based therapy. An expanded pluripotency network described by proteomic assessment of protein-protein interactions between known transcription regulatory factors required for maintenance of ES cells identified combinatorial effects of transcriptional activator complexes required for pluripotency, together with repressor complexes necessary to prevent expression of proteins associated with differentiated cells (47-49).

Stoichiometric correlation between proteins and transcripts can vary (50), and proteomic deconvolution performed in conjunction with other “-omic” studies can enhance systems level analyses (51). For example, in a transcriptome/proteome study that employed chromatin occupancy interrogation with focused epigenomic tracking, discrete transcript and protein changes were revealed to be characteristic of cells released from pluripotency via NANOG depletion (52). This study also demonstrated that epigenetic and post-transcriptional effects targeted distinct subgroups of cellular processes and functions during differentiation, thus emphasizing fine dynamic regulation in and among transcriptome and proteome layers. Furthermore, proteomic characterization of reprogrammed somatic cells (53,54) has significantly identified a highly coordinated biphasic temporal dynamic driving induced pluripotency (55). In another methodological combination study, (metabo)proteomic profiling was used to demonstrate enzymatic restructuring consistent with metabolomic transition from an oxidative to a glycolytic metabolomic phenotype that precedes and guides cell reprogramming (56).

Proteomic studies are also being used to define functional consequences of stem cell-based therapy. For example, extensive proteomic remodeling underlying structural and functional changes associated with onset of dilated cardiomyopathy was reversed by ES cell therapy, with derived protein networks exhibiting a pro-cardiogenic developmental response with concomitant demotion of dysfunctional disease-associated categories (57). Ultimately, the proteomic signature served as a diagnostic of stem cell repair in the setting of dilated cardiomyopathy. Collectively, these studies demonstrate the power of proteomics and of integrative systems biology strategies incorporating proteomics to elucidate molecular properties associated with maintenance or attainment of pluripotency, mechanistic underpinnings of the reprogramming process, and of stem cell therapeutic proteome remodeling in the setting of clinical disease.

## Metabolomics

The metabolome consists of small molecular weight compounds that undergo chemical transformation within the cell. Metabolomics captures the complexity of global metabolism in the context of (patho)physiology, and a multitude of analytical tools have been developed to detect metabolite levels, such as enzymatic analysis, flame ionization, and Raman/Fourier transformed infrared and UV-VIS spectroscopy. Nuclear magnetic resonance (NMR) and mass spectrometry have become methods of choice due to their ability to resolve a wide range of chemical moieties in a high throughput manner (58). The majority of studies to date have utilized targeted approaches to interrogate a defined set of metabolites relevant to a specific biological question. However, with advances in instrumentation, data analysis and compound annotation, broadly inclusive shotgun approaches are routinely employed to reveal a more global profile (59). Resolution of intracellular (fingerprint) and extracellular (footprint) metabolomes has provided insight into metabolic restructuring that guides stem cell differentiation and dedifferentiation (56,60,61). Multiplexing of metabolomics technologies with stable-isotope assessment of metabolic fluxes will further enable dissection of intimate metabolite dynamics to establish metabolic maps defining cell fate.

To bridge the genotype-phenotype continuum, metabolite screening offers a minimally invasive diagnostic approach associated with high patient value and can provide a wealth of information as metabolite profiles serve as functional signatures of enzymatic activity (59). Metabolomic analysis of clinically relevant pathologies enables identification of key metabolites abnormal in identity or quantity. These disease state biomarkers offer fast, reproducible, and cost-effective identification of present or putative disease states (62). Presently, widespread and robust clinical applications of metabolite profiling include screening for inborn errors of metabolism, which is now routinely performed in most of the developed world (63). Targeted tandem mass spectrometry based screening of approximately 30-40 metabolites, with emphasis placed on amino acids and acylcarnitines, now enables diagnosis of over 30 different metabolic disorders, and with greater efficacy than clinical screening alone (64).

A growing number of studies have utilized metabolomic techniques to examine stem cell biology, critical for defining the baseline stem cell metabolic landscape and its perturbation in the diseased state, as well as identifying principal roles of energy metabolism in controlling stem cell fate (65-67). This fundamental work has laid the groundwork for application of stem cell metabolomics as a platform for pharmaceutical toxicity screening and identification of predictive biomarkers of toxicity. Indeed, ES cells treated with valproate were distinguished from vehicle-treated cells based upon a metabolic signature encompassing kynurenine and glutamate metabolism (68-70), demonstrating utility in applying metabolomics deconstruction against an ES cell derived investigative platform, that can ultimately be refined for clinical application.

## Translational application of network biology

Genomic, transcriptomic, proteomic, and metabolomic networks possess unique traits that endow these strata with qualities suitable for use as clinical tools, yet an added level of diagnostic sophistication may be accomplished by leveraging the integrated molecular architecture of these networks against complex disease phenotypes. Network biology, as part of a clinical management strategy, can potentially be employed to identify molecular candidates for pharmacological intervention (71,72). Dissection of molecular interactions presents an opportunity to integrate these “-omic” layers to elucidate the systems etiology of disease occurrence and progression through study of the flow of biological information in and among these layers (73).

Biological networks possess a discriminant set of characteristics that can be quantified (74,75). This information can be used to identify critical genes, proteins, or metabolites in their respective networks that measures not only output of the system, ie, phenotype, but may provide information on biological robustness as well as informational flow (76). Thus, comprehensive molecular cartography through innovative and cutting edge high throughput methodologies can define the functional landscape, or interactome, underlying development and disease (77,78). Furthermore, biological network analysis provides dynamic metrics that permit navigation of this functional topography, serving as a molecular positioning system that identifies features of the interactome critical to maintenance of system function and crucial for targeted interventional translational strategies (9).

## Stem cell informatics: a novel diagnostic paradigm

Systems biology integrates multiple disciplines to create a novel area for translational application, yet faces a plethora of challenges that constrain full implementation. High throughput techniques are expensive and availability to clinical populations at present is financially prohibitive, but as technology improves and assay costs diminish, application of these techniques to the greater clinical population becomes feasible (79). For example, the speed and volume of whole genome sequencing has increased dramatically while concomitantly becoming less expensive in the past decade (80), and continuation of this trend with other “-omic” technologies (81) will ultimately realize translationally applicable personalized bioinformatics (82).

Another parameter associated with high throughput approaches is generation of massive amounts of biodata (83). Logistics of data storage and recovery become critical factors for consideration as a clinically relevant resource, and to address this need, optimization of cloud server storage technology presents an attractive option as a readily accessible and dynamic electronic bioarchive (84). Indeed, continuous development of added-value electronic databases indicates that this growing need to store and parse intricate data sets is actively being addressed (85). It is anticipated that petabytes to exabytes of next generation biodata (86) will emerge in coming years, and for bioinformatic deconvolution to remain practical, cloud-based systems capable of handling these computationally intensive data sets are considered viable platforms to address this need (87). This is particularly critical for temporal profiling that requires integration of molecular data measured over a chronologically ordered series of developmental or disease stages (88,89).

## Tracing molecular signatures from bench to bedside envisioned

**Figure 1:**
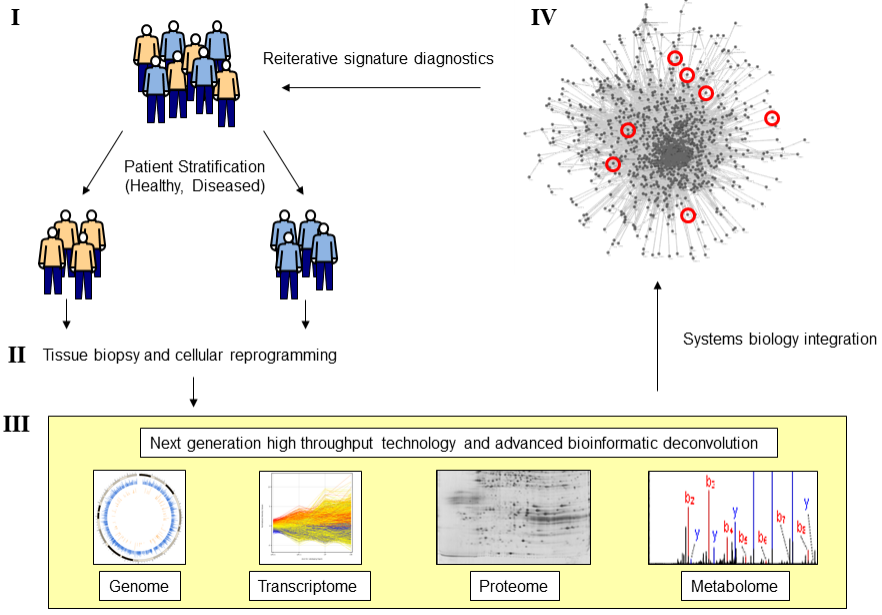
Implementation of advanced diagnostics facilitated by leading generation stem cell informatics. A comprehensive systems biology approach using integrated high throughput screening approaches in combination with the power of reprogrammed (induced pluripotent) stem cells can provide a depth of resolution that can be leveraged against poorly characterized disease etiology. In the illustrated scheme, patient stratification into healthy and diseased cohorts initiates this advanced diagnostic paradigm (**I**). Isolation of cells from patients with diseased tissue can be reprogrammed to an embryonic state (**II**), providing potential zero (embryonic) and end stage (differentiated) time points for longitudinal next generation assays. Generation of individualized and comprehensive multidimensional biological data sets at genome, transcriptome, proteome, and metabolome levels (**III**) can provide advanced clinical resources to track disease progression in real time (systems biology integration) that may be used in construction of an integrated and dynamic network signature (**IV**) to identify novel molecular targets for therapeutic intervention (red circles) in the original patient cohort (reiterative signature diagnostics).

Technology development in the post-genomic area has given rise to advanced high throughput methodologies and modern, integrative bioinformatic tools that permit an unprecedented level of molecular resolution. Dissection of these discrete, yet interrelated molecular strata, in combination with the ability to reprogram phenotypically committed cells to a pluripotent state that provides a unique and patient-specific embryonic pool of cells, enables feasible dynamic and individualized diagnostic strategies for translational application (90-93). For the first time, multiple molecular networks underlying clinical dysfunctions can be mapped and utilized to trace disease etiology (Figure 1). Furthermore, development of a prototype platform for stem cell diagnostics, which incorporates major “-omic” layers discussed here, provides the premise for incorporating other “-omes” for enhanced systems biology deconvolution of disease states. Implementation of this approach as part of a recursive diagnostic algorithm offers the potential for an enhanced modality of patient care made possible by a current medical zeitgeist constructed on an array of novel post-genomic knowledge (94). Ultimately, a collective and dynamic electronic repository for the diversity of biodata constantly generated by leading edge high throughput technology enables powerful meta-level analyses with unmatched precision applicable to multiple disease models (95-98).

Resetting diseased cells to a pluripotent state provides opportunities to track patient-specific changes at primary (genomic), secondary (transcriptomic), tertiary (proteomic), and quaternary (metabolomics) molecular network strata. Construction of an accessible electronic archive, to house large volumes of biodata produced from high grade bioinformatics analyses, is critical for establishing a dynamic clinical resource essential to fully realize comprehensive *ad hoc* diagnosis and real-time molecular tracking of patient pathology and disease progression.
